# Position statement on artificial intelligence (AI) use in evidence synthesis across Cochrane, the Campbell Collaboration, JBI, and the Collaboration for Environmental Evidence 2025

**DOI:** 10.11124/JBIES-25-00480

**Published:** 2025-11-06

**Authors:** Ella Flemyng, Anna Noel-Storr, Biljana Macura, Gerald Gartlehner, James Thomas, Joerg J Meerpohl, Zoe Jordan, Jan Minx, Angelika Eisele-Metzger, Candyce Hamel, Paweł Jemioło, Kylie Porritt, Matthew Grainger

**Affiliations:** 1 Cochrane, UK; 2 Stockholm Environment Institute, Sweden; 3 University for Continuing Education Krems, Austria; 4 EPPI Centre, UCL Social Research Institute, University College London, UK; 5 Institute for Evidence in Medicine, Medical Center – University of Freiburg/Medical Faculty – University of Freiburg, Germany; 6 Cochrane Germany, Cochrane Germany Foundation, Freiburg, Germany; 7 JBI, School of Public Health, The University of Adelaide, Australia; 8 Potsdam Institute for Climate Impacts Research, Germany; 9 Ottawa Hospital Research Institute, Canada; 10 AGH University of Krakow, Poland, and Department of Hygiene and Dietetics; Faculty of Medicine, Jagiellonian University Medical College, Poland; 11 Norwegian Institute for Nature Research, Norway

**KEY MESSAGES**

1. Evidence synthesists are ultimately responsible for their evidence synthesis, including the decision to use artificial intelligence (AI) and automation, and to ensure adherence to legal and ethical standards.
2. Cochrane, the Campbell Collaboration, JBI, and the Collaboration for Environmental Evidence support the aims of the Responsible use of AI in evidence SynthEsis (RAISE) recommendations, which provides a framework for ensuring responsible use of AI and automation across all roles within the evidence synthesis ecosystem.
3. Evidence synthesists developing and publishing syntheses with Cochrane, the Campbell Collaboration, JBI, and the Collaboration for Environmental Evidence can use AI and automation as long as they can demonstrate that it will not compromise the methodological rigor or integrity of their synthesis.
4. AI and automation in evidence synthesis should be used with human oversight.
5. Any use of AI or automation that makes or suggests judgments should be fully and transparently reported in the evidence synthesis report.
6. AI tool developers should proactively ensure their AI systems or tools adhere to the RAISE recommendations so we have clear, transparent, and publicly available information to inform decisions about whether an AI system or tool could and should be used in evidence synthesis.

Evidence syntheses, including systematic reviews, are a type of research that uses systematic, replicable methods to evaluate all available evidence on a specific question. They are built on the principles of research integrity, including rigor, transparency, and reproducibility. There is wide recognition that artificial intelligence (AI) and automation have the potential to transform the way we produce evidence syntheses, making the process significantly more efficient. However, this technology is potentially disruptive, characterized by opaque decision making and black-box predictions, susceptible to overfitting, potentially embedded with algorithmic biases, and at risk of fabricated outputs and hallucinations. To safeguard evidence synthesis as the cornerstone of trusted, evidence-informed decision making, Cochrane, the Campbell Collaboration, JBI, and the Collaboration for Environmental Evidence (CEE) have come together to collaborate on a responsible and pragmatic approach to AI use in evidence synthesis.

By AI, we mean different types of automation, as described within the Responsible use of AI in evidence SynthEsis recommendations (RAISE)^1^; specifically, “advanced technologies that enable machines to do highly complex tasks effectively—which would require intelligence if a person were to perform them.” This ranges from general automation applications, such as rule-based or trained machine learning algorithms, to more recent large language models and generative AI approaches.

Incorporating AI in evidence synthesis comes with challenges as well as opportunities. Whilst it is clear we need to make better use of AI for evidence synthesis to become more timely, affordable, and sustainable, we must also acknowledge the environmental and social costs associated with some forms of AI, particularly large-scale language models. There are risks that misuse could erode methodological standards by exacerbating existing biases and reducing reliability.^2^,^3^ These concerns are particularly relevant as current AI developments are often driven by commercial interests, and as such are often opaque regarding limitations, and lacking appropriate validation and evaluation. Overall, this undermines the reliability and replicability of AI-driven outputs.

To this end, Cochrane, the Campbell Collaboration, JBI, and the CEE have come together to form a joint AI Methods Group.^4^ The group officially supports the aims of RAISE,^1^ which states that we need to work together to ensure AI does not compromise the principles of research integrity on which evidence synthesis was built. RAISE offers tailored recommendations for roles across the evidence synthesis ecosystem: from evidence synthesists to methodologists; from AI development teams to organizations or publishers involved in evidence synthesis. It is a first step to help clarify everyone’s responsibilities to ensure safe and responsible AI use. Whilst each of our organizations has different resources and infrastructure, and therefore our approach to implementing the RAISE recommendations may differ, our aim is to align with best practice principles and share lessons learned on effective approaches.

RAISE, and our position on AI use for those who author evidence synthesis with our organizations (Table 1), will continue to evolve as the field and evidence base evolves. We recommend that the most up-to-date version of RAISE^1^ and this published position statement should always be used.

**Table 1:** Cochrane, the Campbell Collaboration, JBI, and the Collaboration for Environmental Evidence position for evidence synthesists on AI use based on Responsible use of AI in evidence SynthEsis (RAISE) recommendations (version 2.1 in development as of 22 September 2025)

RAISE recommendation	Further guidance
Remain ultimately responsible for the evidence synthesis
	1. An author is accountable for the content, methods, and findings of their evidence synthesis, including the decision to use AI, how it is used, and its impact on the synthesis.
	2. When considering using an AI system or tool, be critical of its evaluations,^5^ to understand whether it does what it states to an adequate level, as well as its limitations and whether it can be applied to the context of the specific synthesis.^6^
	3. Use of AI should be justified and should involve demonstrating that the tools are methodologically sound, that they do not undermine the trustworthiness or reliability of the synthesis or its conclusions, and that it is appropriate to use a specific AI system or tool in the context of the specific evidence synthesis.
Report AI use in your evidence synthesis manuscript transparently
	1. Authors can use AI within their syntheses and to prepare their manuscript.^7^-^10^
	2. Authors should declare when they have used AI if it makes or suggests judgments, such as in relation to the eligibility of a study, appraisals (including risk of bias assessments), extraction of bibliographic, numerical, or qualitative data from a study or its results, synthesis of data from two or more studies, assessments of the certainty of evidence (including GRADE domains or overall certainty ratings for an outcome or finding), drafting text that summarizes the overall strength of evidence, related implications for decision making or research, or plain language summaries. Generally, AI used to improve spelling, grammar, or manuscript structure does not need to be listed, but we recommend authors check the journal’s specific policy to ensure adherence.
	3. Adhere to the established reporting standards used by each journal, such as PRISMA^11^ or ROSES.^12^ PRISMA, for example, includes items on reporting automation tools used at different stages of the synthesis process. This should be reported in the section specified by each journal, such as Acknowledgments, Methods, or a specific section for disclosure of AI use. If these details are extensive or the AI is used in multiple stages of the synthesis process, consider using supplementary materials or tabular presentation, or both. In general, authors should report the following:
	a. The name(s) of the AI system(s), tool(s) or platform(s), version(s) and date(s) used.
	b. The purpose of using AI and which parts of the evidence synthesis process were impacted. Cite or reference user guidance or report how AI was used, including any modifications that were applied.
	c. The justification for using AI, including evidence that the AI system or tool is methodologically sound and will not undermine the trustworthiness or reliability of the synthesis or its conclusions (eg, citing or referencing evaluations of its performance that detail the impact of errors, limitations, and generalizability) and how it has been validated (and piloted, if applicable) to ensure that it is appropriate for use in the context of the specific evidence synthesis. Wherever possible and practical, make the inputs (eg, prompt development), outputs, datasets, and code publicly and freely available (for instance, on repositories or as supplementary materials) and describe any steps taken to verify AI-generated outputs.
	d. Any financial and non-financial interests the evidence synthesists have in the AI system or tool, along with the AI system or tool’s funding sources.
	e. Any limitations of using AI in the review processes, including any potential biases. Comment on the potential impact of each limitation.
Ensure ethical, legal, and regulatory standards are adhered to when using AI	Ensure ethical, legal, and regulatory standards are adhered to as part of applying AI to your synthesis. For example, be aware of issues relating to plagiarism, provenance, copyright, intellectual property, jurisdiction, licensing; and confidentiality, compliance, and privacy responsibilities, including data protection laws.^6^

A key area for further guidance is how to approach justifying the use of AI in the specific synthesis. During protocol development, evidence synthesists need to consider many potential trade-offs, often related to balancing capacity and resource availability against questions of urgency, relevance, and scope. It can be helpful to consider the use of AI as an additional trade-off decision you need to make. As part of the decision, evidence synthesists should consider the context of their synthesis (eg, who will use it and what it is for), what the risk tolerance might be for errors affecting the findings or conclusions of the synthesis, and what potential risk mitigation strategies are available.

In some contexts, the use of AI may enhance the quality of evidence synthesis and help address some of the trade-offs inherent to accelerated timelines. Cochrane’s Rapid Review Methods Group has a separate position statement on this (currently under editorial consideration). For example, abstract screening in rapid reviews is often conducted by a single review author, which carries an estimated 13% risk of falsely excluding a relevant study.^13^ Using AI as a second “reviewer” could help reduce this risk.

For evidence synthesists to be able to make informed decisions, AI tool developers must ensure that there is publicly available and transparent information about the AI system or tools.^6^ Our organizations therefore call for AI tool developers to adhere proactively to the RAISE recommendations,^1^ in particular, ensuring there is:

1. clear, public information about how the AI system or tool works, and terms and conditions;
2. publicly available testing, training, and validation evaluations with full and transparent reporting, including the scope, domain, sources, methods, etc., of the data used in each^5^; and
3. public and transparent information on the strengths and limitations of the AI system or tool, including any potential biases, which relates to the scope, domains, and breadth of the data used for training, testing, and validation.

Evaluation and validation studies are critical to determine whether an AI system or tool performs to an adequate level for evidence syntheses in general, as well as for the specific topics addressed in a given synthesis. Although AI tool developers are responsible for assessing their tools and systems, evaluations by independent methodologists who do not have a conflict of interest in their performance should also be considered whenever available. Clear terms and conditions are vital for ensuring evidence synthesists can adhere to ethical, legal, and regulatory standards. Information about the system and the scope and domains of the data used for training, testing, and validation will help evidence synthesists understand the strengths and limitations, and how well it will apply to their synthesis. For example, the more similar the scope and domains are to your synthesis, the more confidence you could have in using it. Biases may include using English-only or open-access-only data in the training and testing stages, which may or may not be important to the synthesis context. Alternatively, the appropriately justified use of an AI system or tool in another similar scoped evidence synthesis could form the basis of your justification to use it. Depending on how certain an evidence synthesist is about an AI system or tool in their synthesis, they may need to pilot (or calibrate) the AI system or tool to validate its performance within their evidence synthesis, to ensure its use will not undermine the trustworthiness or reliability of the synthesis or its conclusions. This requires upfront investment from the author team in terms of time, effort, and skill, so a decision will need to be made as to whether the effort is worth the potential gain.

This process to consider the trade-off is the foundation for ensuring human oversight in the decision and application of AI in evidence synthesis. Work is under way to develop a framework that guides evidence synthesists through these considerations, so decisions can be made transparently and consistently.

Decisions to use AI should be considered and reported as part of protocol development. Below is a generic reporting template that could be used:

We will use [*AI system/tool/approach name, version, date*] developed by [*organization/developer*] for [*specific purpose(s)*] in [*the evidence synthesis process*]. The [*AI system/tool/approach*] will [*state it will be used according to the user guide, and include reference, and/or briefly describe any customization, training, or parameters to be applied*]. Outputs from the [*AI system/tool/approach*] are justified for use in our synthesis because [*describe how you have determined it is methodologically sound and will not undermine the trustworthiness or reliability of the synthesis or its conclusions and how it has been validated or calibrated to ensure that it is appropriate for use in the context of the specific evidence synthesis, if not covered in the user guide, evaluations, or elsewhere*]. Limitations [*of the AI system/tool/approach*] include [*describe known limitations, potential biases, and ethical concerns]/[are included as a supplementary material*]. [*If applicable*] A detailed description of the methodology, including parameters and validation procedures, is available in [*supplementary materials*].

We understand that this is a new frontier for evidence synthesists to navigate. How do you know when it is appropriate and safe to use AI in your synthesis? In addition to the recommendations for the different roles in the evidence synthesis ecosystem, RAISE also includes guidance for implementing the recommendations in practice, covering building and validation of AI systems and tools, conducting evaluations to build a cumulative evidence base for AI use, performance metrics for AI evaluations, the current status of available AI for evidence synthesis, selecting and using AI in your evidence synthesis, and ethical, legal, and regulatory considerations when using AI.^1^,^5^,^6^

Ultimately, evidence synthesists are responsible for their work, how they use AI, and any implications, including social and environmental impacts. Our organizations and the joint Methods Group are committed to improving AI literacy for our authors and editors to help make these decisions around AI use. We are aligning our work with developments happening across the ecosystem, including the Evidence Synthesis Infrastructure Collaborative,^14^ the RAISE initiative,^1^ and the Digital Evidence Synthesis Tool INnovation for Yielding Improvements in Climate & Health (DESTINY) project,^15^ among others. This is to ensure that the members of Cochrane, the Campbell Collaboration, JBI, and CEE, as well as the wider field, have the resources and guidance they need to use AI responsibly, efficiently, and equitably within their evidence synthesis.

## Declarations of interest

All authors are leaders in the joint AI Methods Group with Cochrane, the Campbell Collaboration, JBI, and the Collaboration for Environmental Evidence. EF and ANS are employed by Cochrane. ZJ and KP are employed by JBI. BM and MG are associated with the Collaboration for Environmental Evidence. GG is associated with Cochrane Austria. JM and AEM are associated with Cochrane Germany. JM is associated with Campbell Climate Solutions. PJ is associated with Cochrane Poland. JT, EF, ANS, ZJ, PJ, BM, JM, JJM, GG are authors of the Responsible use of AI in evidence SynthEsis recommendations. No authors listed were involved in the editorial process or decisions for this editorial.

## Acknowledgments

This article has been published simultaneously with *JBI Evidence Synthesis, Campbell Systematic Reviews, Cochrane Database of Systematic Reviews,* and *Environmental Evidence*. We would like to include a special thanks for the detailed feedback from the following groups that informed this statement: Cochrane Editorial Board, including patient representatives; Cochrane Methods Executive; Campbell Executive editor team; JBI Executive team; and the Collaboration for Environmental Evidence Executive team.

## Provenance and peer review

This editorial was commissioned based on a co-publication with *JBI Evidence Synthesis, Campbell Systematic Reviews, Cochrane Database of Systematic Reviews,* and *Environmental Evidence*. This position statement was fully peer reviewed by leaders of Cochrane, the Campbell Collaboration, JBI, and the Collaboration for Environmental Evidence as part of the endorsement process.
